# Impact of Impaired Renal Function on the Efficacy and Safety of Second‐Line Tyrosine Kinase Inhibitor Therapy After First‐Line Immuno‐Oncology Combination Therapy in Metastatic Renal Cell Carcinoma: A Japanese Multicenter Retrospective Study

**DOI:** 10.1111/iju.70172

**Published:** 2025-07-04

**Authors:** Naoki Fujita, Yuto Matsushita, Takahiro Kojima, Yukari Bando, Takahiro Osawa, Tomokazu Sazuka, Keisuke Goto, Kazuyuki Numakura, Kazutoshi Yamana, Shuya Kandori, Yoshihide Kawasaki, Takuma Kato, Makito Miyake, Kazutoshi Fujita, Kosuke Ueda, Hajime Tanaka, Ryotaro Tomida, Hiroshi Kitamura, Hideaki Miyake, Shingo Hakakeyama, the Japanese Urological Oncology Group

**Affiliations:** ^1^ Department of Urology Hirosaki University Graduate School of Medicine Hirosaki Japan; ^2^ Department of Urology Hamamatsu University School of Medicine Hamamatsu Japan; ^3^ Department of Urology Aichi Cancer Center Nagoya Japan; ^4^ Division of Urology Kobe University Graduate School of Medicine Kobe Japan; ^5^ Department of Urology Hokkaido University Hospital Sapporo Japan; ^6^ Department of Urology Graduate School of Medicine, Chiba University Chiba Japan; ^7^ Department of Urology Hiroshima University Graduate School of Biomedical and Health Sciences Hiroshima Japan; ^8^ Department of Urology Akita University Graduate School of Medicine Akita Japan; ^9^ Department of Urology and Molecular Oncology Niigata University Graduate School of Medical and Dental Sciences Niigata Japan; ^10^ Department of Urology, Institute of Medicine University of Tsukuba Tsukuba Japan; ^11^ Department of Urology Tohoku University Graduate School of Medicine Sendai Japan; ^12^ Department of Urology, Faculty of Medicine Kagawa University Takamatsu Kagawa Japan; ^13^ Department of Urology Nara Medical University Kashihara Japan; ^14^ Department of Urology Kindai University Faculty of Medicine Osaka Japan; ^15^ Department of Urology Kurume University School of Medicine Kurume Japan; ^16^ Department of Urology Institute of Science Tokyo Tokyo Japan; ^17^ Department of Urology Tokushima University Graduate School of Biomedical Sciences Tokushima Japan; ^18^ Department of Urology, Faculty of Medicine University of Toyama Toyama Japan

**Keywords:** chronic kidney disease, oncological outcomes, renal function decline, safety, second‐line TKI

## Abstract

**Objectives:**

To evaluate the effects of renal impairment at the time of second‐line tyrosine kinase inhibitor (TKI) therapy initiation and rapid renal function decline during first‐line immuno‐oncology (IO) combination therapy on metastatic renal cell carcinoma (mRCC) patients treated with second‐line TKIs.

**Methods:**

This multicenter retrospective study included 243 mRCC patients treated with first‐line IO combination therapy, followed by second‐line TKI therapy. Patients were divided into three groups using the estimated glomerular filtration rate (eGFR; mL/min/1.73 m^2^) at the time of second‐line TKI therapy initiation: eGFR ≥ 60, 30 ≤ eGFR < 60, and eGFR < 30. The eGFR slope during first‐line IO combination therapy was calculated using eGFR measurements when initiating first‐line and second‐line therapies. Multivariable Cox proportional hazards regression analyses were performed to evaluate the effects of renal impairment and eGFR slope on progression‐free survival (PFS) and overall survival (OS).

**Results:**

The incidence rates of any grade and grade ≥ 3 adverse events were not significantly different among the three groups. Univariable analyses indicated that eGFR slope was not significantly associated with PFS or OS. Multivariable analyses suggested that moderate (30 ≤ eGFR < 60 mL/min/1.73 m^2^) and severe (eGFR < 30 mL/min/1.73 m^2^) renal impairment had no effects on shorter PFS, whereas severe renal impairment was independently and significantly associated with shorter OS.

**Conclusions:**

TKIs can be safely used as a second‐line treatment after first‐line IO combination therapy in mRCC patients with renal impairment without sacrificing oncological outcomes, except for in patients with severe renal impairment.

**Abbreviations**
AEs: adverse events
CKD: chronic kidney disease
ECOG PS: Eastern Cooperative Oncology Group performance status
eGFR: estimated glomerular filtration rate
IMDC: International Metastatic RCC Database Consortium
IO: immuno‐oncology
JUOG: Japanese Urological Oncology Group
mRCC: metastatic renal cell carcinoma
ORR: objective response rate
OS: overall survival
PFS: progression‐free survival
RCC: renal cell carcinoma
TKIs: tyrosine kinase inhibitors

## 1 Introduction

Approximately 20%–30% of patients with renal cell carcinoma (RCC) present with metastatic disease at the initial diagnosis [1]. After the discovery of immune regulatory mechanisms [2], the treatment paradigm for metastatic RCC (mRCC) has shifted toward immuno‐oncology (IO) combination therapy, leading to significant improvements in prognosis [3].

Tyrosine kinase inhibitors (TKIs) are one of the therapeutic options as second‐line treatment following first‐line IO combination therapy. Although several studies have reported the safety and efficacy of second‐line TKIs after first‐line IO combination therapy [4, 5, 6, 7, 8], the specific factors influencing these parameters in this setting remain unclear.

RCC patients often have chronic kidney disease (CKD) from the tumor itself, surgical loss of the kidney parenchyma, and acute kidney injury occurring during surgical and drug treatments [9, 10]. Despite such a high prevalence of CKD, few data are available about the safety and efficacy of TKIs in patients with CKD, especially after first‐line IO combination therapy [11, 12, 13]. Moreover, IO therapies can cause gradual renal function decline over time [14], with rapid renal function decline reportedly associated with an increased risk of cancer‐specific mortality in several malignancies [15, 16]. However, no study has investigated the association between rapid renal function decline during first‐line IO combination therapy and oncological outcomes in patients with mRCC.

Thus, the aim of the present study was to evaluate the effects of CKD at the time of second‐line TKI therapy initiation and rapid renal function decline during first‐line IO combination therapy on oncological outcomes in patients with mRCC who were treated with second‐line TKIs.

## 2 Methods

### 2.1 Ethics Statement

This retrospective multicenter study was conducted under the Japanese Urological Oncology Group (JUOG) framework and approved by the ethics review board at Hamamatsu University School of Medicine (Approval No. 22‐008), followed by those of all 34 participating institutions in Japan. The need to obtain informed consent was waived because of the retrospective design; however, the ability to opt out was provided through the websites of all institutions.

### 2.2 Patient Selection

This study included 243 Japanese patients who were diagnosed with mRCC and treated with first‐line IO combination therapy, followed by second‐line TKI therapy, between August 2018 and January 2022 at 34 institutions belonging to the JUOG. The first‐line IO combination therapy included nivolumab plus ipilimumab, pembrolizumab plus axitinib, and avelumab plus axitinib.

Renal function was evaluated by the estimated glomerular filtration rate (eGFR) using a modified version of the abbreviated Modification of Diet in Renal Disease Study formula for Japanese participants at the time of second‐line TKI therapy initiation. CKD was defined as eGFR < 60 mL/min/1.73 m^2^. Patients were divided into three groups: those with an eGFR ≥ 60 mL/min/1.73 m^2^ (eGFR ≥ 60 group), eGFR < 60 and ≥ 30 mL/min/1.73 m^2^ (30 ≤ eGFR < 60 group), and eGFR < 30 mL/min/1.73 m^2^ (eGFR < 30 group).

### 2.3 Evaluation of Variables

The following data were collected from the medical records: age, sex, Eastern Cooperative Oncology Group performance status (ECOG PS), histology, sarcomatoid component, history of nephrectomy, first‐line combination therapy regimens, second‐line TKI therapy agents, International Metastatic RCC Database Consortium (IMDC) risk, metastatic organs, and laboratory values, including hemoglobin, neutrophil, platelet, corrected calcium, lactate dehydrogenase, albumin, C‐reactive protein, and eGFR levels. Age, ECOG PS, IMDC risk, metastatic organs, and laboratory values were evaluated at the time of second‐line TKI therapy initiation. The eGFR slope during first‐line IO combination therapy was calculated using the eGFR measurements at the time of first‐line and second‐line therapy initiation as follows: (eGFR at the time of second‐line therapy initiation—eGFR at the time of first‐line therapy initiation [mL/min/1.73 m^2^])/duration of first‐line therapy (years). All adverse events (AEs) that occurred during second‐line TKI therapy were collected.

### 2.4 Statistical Analysis

SPSS (version 24.0; IBM Corp., Armonk, NY, USA) and GraphPad Prism 5.03 (GraphPad Software, San Diego, CA, USA) were used to perform the statistical analyses. Differences in quantitative variables between two groups were analyzed using the Mann–Whitney *U* test. Categorical variables were compared using the Fisher's exact test or chi‐squared test. The changes in eGFR from first‐line therapy initiation to second‐line therapy initiation were analyzed using the paired *t*‐test. The best response was assessed according to the Response Evaluation Criteria in Solid Tumors v1.1, with an objective response defined as complete response or partial response. Progression‐free survival (PFS) and overall survival (OS) were evaluated using the Kaplan–Meier method and compared using the log‐rank test. Univariable and multivariable Cox proportional hazards regression analyses were performed to evaluate the effects of renal impairment and eGFR slope on PFS and OS. These outcomes were calculated from the date of second‐line TKI therapy initiation to the date of the first event or last follow‐up. Statistical significance was set at *p* < 0.05.

## 3 Results

### 3.1 Patient Background

The median age and follow‐up period after second‐line TKI therapy initiation were 53 years and 12 months, respectively. The first‐line regimens are presented in Table 1. As the second‐line therapy, 118 (49%), 92 (38%), 23 (9.5%), nine (3.7%), and one (0.4%) were treated with cabozantinib, axitinib, pazopanib, sunitinib, and sorafenib, respectively (Table 1). Of the 243 patients, 53 (22%) had non‐clear cell RCC.

**TABLE 1:** Patient background at the time of second‐line therapy initiation.

	eGFR ≥ 60 group	30 ≤ eGFR < 60 group	eGFR < 30 group	*p* ^a^	*p* ^b^	*p* ^c^
	*n* = 107	*n* = 112	*n* = 24			
Age, years	63 (52–67)	70 (65–75)	74 (66–77)	< 0.001	< 0.001	0.153
Male	84 (79%)	83 (74%)	19 (79%)	0.445	1.000	0.796
ECOG PS	0.0 (0.0–1.0)	0.0 (0.0–1.0)	1.0 (0.0–1.0)	0.509	0.187	0.352
mRCC type				0.728	0.978	0.812
De novo	74 (69%)	75 (67%)	16 (67%)			
Recurrent	33 (31%)	37 (33%)	8 (33%)			
Prior nephrectomy	55 (51%)	69 (62%)	14 (58%)	0.128	0.539	0.765
Cytoreductive nephrectomy	21 (20%)	28 (25%)	2 (8.3%)	0.340	0.245	0.103
Histology				0.395	0.732	0.388
Clear cell	81 (76%)	84 (75%)	17 (71%)			
Non‐clear cell	24 (22%)	22 (20%)	7 (29%)			
Unknown	2 (1.9%)	6 (5.4%)	0 (0.0%)			
Sarcomatoid component				0.895	0.892	0.904
Present	11 (10%)	11 (9.8%)	3 (13%)			
Absent	90 (84%)	93 (83%)	20 (83%)			
Unknown	6 (5.6%)	8 (7.1%)	1 (4.2%)			
IMDC risk at the time of first‐line therapy				0.716	0.694	0.480
Favorable	13 (12%)	17 (15%)	1 (4.2%)			
Intermediate	65 (61%)	60 (54%)	16 (67%)			
Poor	28 (26%)	33 (29%)	7 (29%)			
Unknown	1 (0.9%)	2 (1.8%)	0 (0.0%)			
First‐line therapy				0.931	0.319	0.410
Ipilimumab + Nivolumab	81 (76%)	88 (79%)	20 (83%)			
Pembrolizumab + Axitinib	21 (20%)	19 (17%)	2 (8.3%)			
Avelumab + Axitinib	5 (4.7%)	5 (4.5%)	2 (8.3%)			
Reason for first‐line therapy discontinuation				0.943	0.632	0.661
Progressive disease	75 (70%)	79 (71%)	18 (75%)			
AE or unknown	32 (30%)	33 (29%)	6 (25%)			
Duration of first‐line therapy, months	5.9 (3.4–9.9)	7.5 (4.9–11.6)	6.6 (4.2–11.7)	0.010	0.430	0.516
IMDC risk at the time of second‐line therapy				0.782	0.179	0.196
Favorable	14 (13%)	13 (12%)	0 (0.0%)			
Intermediate	59 (55%)	67 (60%)	15 (63%)			
Poor	34 (32%)	32 (29%)	9 (38%)			
Metastatic site at the time of second‐line therapy						
Lungs	74 (69%)	80 (71%)	15 (63%)	0.713	0.528	0.387
Liver	23 (22%)	18 (16%)	8 (33%)	0.304	0.218	0.051
Bone	50 (47%)	32 (29%)	10 (42%)	0.006	0.653	0.208
Brain	6 (5.6%)	7 (6.3%)	1 (4.2%)	0.841	1.000	1.000
Contralateral kidney after nephrectomy	6 (5.6%)	6 (5.4%)	1 (4.2%)	0.935	1.000	1.000
Local recurrence after nephrectomy	8 (7.5%)	8 (7.1%)	1 (4.2%)	0.924	1.000	1.000
Laboratory blood test						
LDH, IU/L	197 (169–280)	207 (168–266)	251 (180–323)	0.841	0.174	0.168
Albumin, g/dL	3.8 (3.1–4.2)	3.8 (3.2–4.1)	3.3 (2.6–3.8)	0.497	0.009	0.025
CRP, mg/dL	1.1 (0.2–3.8)	0.7 (0.1–3.4)	1.3 (0.1–8.8)	0.263	0.726	0.430
eGFR, mL/min/1.73 m^2^	78 (66–93)	45 (38–51)	17 (8.3–27)	< 0.001	< 0.001	< 0.001
Second‐line therapy				0.812	0.893	0.820
Cabozantinib	54 (50%)	53 (47%)	11 (46%)			
Axitinib	39 (36%)	42 (38%)	11 (46%)			
Pazopanib	11 (10%)	10 (8.9%)	2 (8.3%)			
Sunitinib	3 (2.8%)	6 (5.4%)	0 (0.0%)			
Sorafenib	0 (0.0%)	1 (0.9%)	0 (0.0%)			
Follow‐up period, months	14 (7.6–23)	12 (8.0–19)	6.0 (4.5–16)			

*Note:* All data are presented as *n* (%) or median (interquartile range).

Abbreviations: AE, adverse event; CRP, C‐reactive protein; ECOG PS, Eastern Cooperative Oncology Group performance status; eGFR, estimated glomerular filtration rate; IMDC, International Metastatic RCC Database Consortium; LDH, lactate dehydrogenase; mRCC, metastatic renal cell carcinoma.

^a^ eGFR ≥ 60 group vs. 30 ≤ eGFR < 60 group.

^b^ eGFR ≥ 60 group vs. eGFR < 30 group.

^c^ 30 ≤ eGFR < 60 group vs. eGFR < 30 group.

The median eGFR at the time of second‐line TKI therapy initiation in all patients was 53.8 mL/min/1.73 m^2^. Of the 243 patients, 107 (44%), 112 (46%), and 24 (9.9%) were classified into the eGFR ≥ 60, 30 ≤ eGFR < 60, and eGFR < 30 groups, respectively. In the eGFR < 30 group, five (21%) patients were undergoing dialysis at the time of second‐line TKI therapy initiation. Among the three groups, significant differences were found in the patients' backgrounds for age, bone metastasis, albumin values, and renal function (Table 1).

### 3.2 AEs Associated With Second‐Line TKI Therapy

**FIGURE 1:**
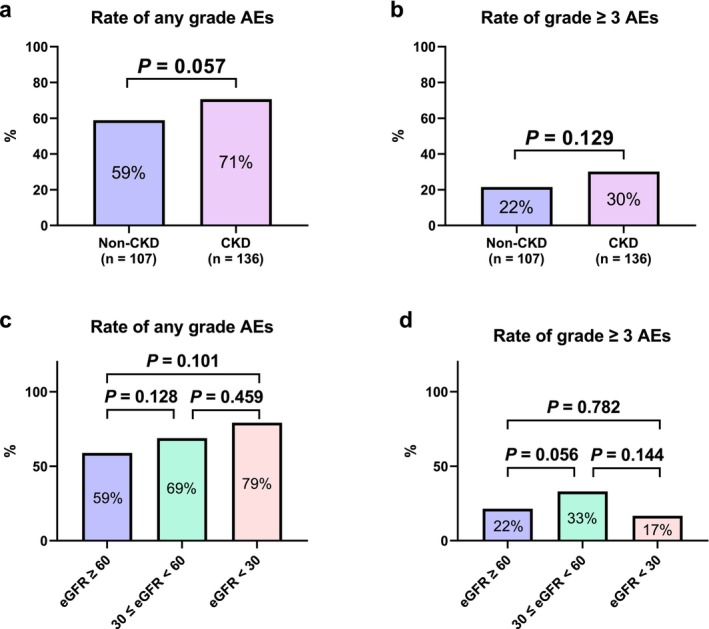
Adverse events (AEs) associated with second‐line tyrosine kinase inhibitor therapy. The incidence rates of any grade (a) and grade ≥ 3 AEs (b) were compared between patients with and without chronic kidney disease (CKD) using the chi‐squared test. The incidence rates of any grade (c) and grade ≥ 3 AEs (d) were compared among the three estimated glomerular filtration rate (eGFR) groups using the Fisher's exact test or chi‐squared test.

The incidence rates of any grade AEs and grade ≥ 3 AEs in all patients were 65% and 26%, respectively. These AE incidence rates were not significantly different between patients with and without CKD (Figure 1a,b), nor were they significantly different among the eGFR ≥ 60, 30 ≤ eGFR < 60, and eGFR < 30 groups (Figure 1c,d).

### 3.3 Associations Between Renal Function Decline During First‐Line IO Combination Therapy and Oncological Outcomes

**FIGURE 2:**
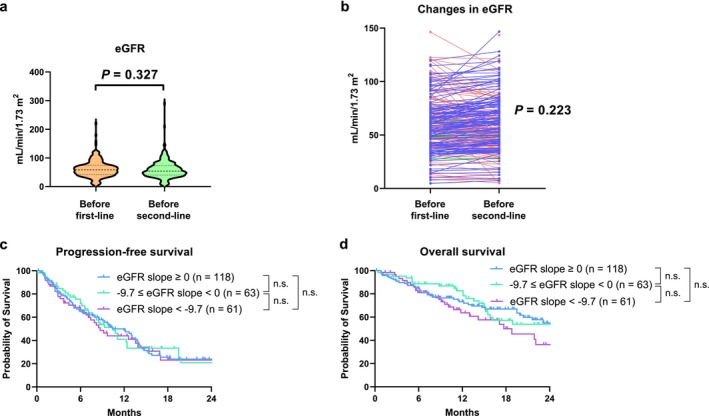
Associations between renal function decline during first‐line therapy and oncological outcomes. The estimated glomerular filtration rate (eGFR) was compared at the time of first‐line therapy initiation and second‐line therapy initiation using the Mann–Whitney *U* test (a). The changes in eGFR from first‐line therapy initiation to second‐line therapy initiation were compared using the paired *t*‐test (b). The blue, green, and red lines respectively indicate an increase, no change, and decrease in eGFR during first‐line therapy. Progression‐free survival (c) and overall survival (d) were evaluated using the Kaplan–Meier method and compared using the log‐rank test. These outcomes were calculated from the date of second‐line tyrosine kinase inhibitor therapy initiation to the date of the first event or last follow‐up. n.s., not significant.

No significant difference in eGFR was observed at the time of first‐line therapy initiation compared with second‐line therapy initiation (Figure 2a; median values: 58 and 54 mL/min/1.73 m^2^, respectively; *p* = 0.327). When patients were stratified by prior nephrectomy status, similar results were observed between those who had undergone nephrectomy (Figure S1a) and those who had not (Figure S1b). Likewise, stratification by their first‐line regimen showed similar outcomes between patients treated with the IO‐IO combination (Figure S1c) and IO plus TKI therapies (Figure S1d). The median change in eGFR from first‐line therapy initiation to second‐line therapy initiation in all patients was 0.0 mL/min/1.73 m^2^ (interquartile range [IQR]: −8.1–4.6 mL/min/1.73 m^2^), which was not significantly different (Figure 2b; *p* = 0.223). Of the 243 patients, 118 (49%) experienced renal function decline in any degree during first‐line IO combination therapy. The median eGFR slope in all patients was 0.0 mL/min/1.73 m^2^ per year (IQR: −9.7–9.7 mL/min/1.73 m^2^ per year). When the patients were divided according to their median and third quartile eGFR slope values, PFS was not significantly different between patients with eGFR slope ≥ 0.0 mL/min/1.73 m^2^ per year and eGFR slope < 0.0 and ≥ −9.7 mL/min/1.73 m^2^ per year (*p* = 0.917), between eGFR slope < 0.0 and ≥ −9.7 mL/min/1.73 m^2^ per year and eGFR slope < −9.7 mL/min/1.73 m^2^ per year (*p* = 0.664), or between eGFR slope ≥ 0.0 mL/min/1.73 m^2^ per year and eGFR slope < −9.7 mL/min/1.73 m^2^ per year (*p* = 0.717) (Figure 2c). When the patients were stratified by their first‐line regimens, similar results were observed among those treated with the IO‐IO combination (Figure S1e) and IO plus TKI therapies (Figure S1f) as the first‐line therapy. OS was not significantly different between patients with eGFR slope ≥ 0.0 mL/min/1.73 m^2^ per year and eGFR slope < 0.0 and ≥ −9.7 mL/min/1.73 m^2^ per year (*p* = 0.918), between eGFR slope < 0.0 and ≥ −9.7 mL/min/1.73 m^2^ per year and eGFR slope < −9.7 mL/min/1.73 m^2^ per year (*p* = 0.146), or between eGFR slope ≥ 0.0 mL/min/1.73 m^2^ per year and eGFR slope < −9.7 mL/min/1.73 m^2^ per year (*p* = 0.124) (Figure 2d). The univariable analyses suggested that eGFR slope was not significantly associated with PFS or OS (Tables 2 and 3).

**TABLE 2:** Univariable analyses for progression‐free survival.

	Factor	*p*	Hazard ratio	95% CI
Age	Continuous	0.178	0.989	0.973–1.005
Sex	Male	0.099	0.739	0.516–1.058
Prior nephrectomy	Performed	0.007	0.636	0.458–0.882
Histopathological type	Non‐clear cell	< 0.001	2.040	1.397–2.979
Sarcomatoid component	Present	0.080	1.600	0.945–2.708
First‐line therapy	IO plus TKI	0.685	1.087	0.726–1.628
Reason for first‐line treatment discontinuation	Progressive disease	0.011	1.691	1.128–2.536
Lactate dehydrogenase^a^	Continuous	0.039	1.000	1.000–1.001
Albumin^a^	Continuous	< 0.001	0.580	0.456–0.739
C‐reactive protein^a^	Continuous	< 0.001	1.095	1.065–1.125
Lung metastasis^a^	Present	0.218	1.259	0.873–1.816
Liver metastasis^a^	Present	0.047	1.479	1.004–2.179
Bone metastasis^a^	Present	0.240	1.221	0.875–1.705
Brain metastasis^a^	Present	0.995	1.002	0.510–1.969
Contralateral renal metastasis^a^	Present	0.633	1.191	0.581–2.439
Local recurrence^a^	Present	0.513	1.240	0.652–2.359
Second‐line therapy	Cabozantinib	0.522	0.898	0.645–1.249
IMDC risk^a^	Favorable	Reference		
	Intermediate	0.037	2.015	1.045–3.884
	Poor	< 0.001	3.881	1.961–7.683
eGFR slope	≥ 0 mL/min/1.73 m^2^ per year	Reference		
	< 0 and ≥ −9.7 mL/min/1.73 m^2^ per year	0.896	0.974	0.655–1.447
	< −9.7 mL/min/1.73 m^2^ per year	0.711	1.079	0.723–1.609
eGFR^a^	≥ 60 mL/min/1.73 m^2^	Reference		
	< 60 and ≥ 30 mL/min/1.73 m^2^	0.722	0.939	0.664–1.328
	< 30 mL/min/1.73 m^2^	0.041	1.776	1.024–3.081

Abbreviations: CI, confidence interval; eGFR, estimated glomerular filtration rate; IMDC, International Metastatic RCC Database Consortium; IO, immuno‐oncology; TKI, tyrosine kinase inhibitor.

^a^ These variables were evaluated at the time of second‐line therapy initiation.

**TABLE 3:** Univariable analyses for overall survival.

	Factor	*p*	Hazard ratio	95% CI
Age	Continuous	0.948	0.999	0.980–1.019
Sex	Male	0.070	0.671	0.436–1.033
Prior nephrectomy	Performed	0.004	0.547	0.364–0.821
Histopathological type	Non‐clear cell	0.006	1.867	1.194–2.921
Sarcomatoid component	Present	0.043	1.840	1.019–3.320
First‐line therapy	IO plus TKI	0.937	1.020	0.616–1.692
Reason for first‐line treatment discontinuation	Progressive disease	0.004	2.256	1.294–3.934
Lactate dehydrogenase^a^	Continuous	0.249	1.000	1.000–1.001
Albumin^a^	Continuous	< 0.001	0.370	0.276–0.495
C‐reactive protein^a^	Continuous	< 0.001	1.120	1.088–1.154
Lung metastasis^a^	Present	0.806	1.057	0.679–1.645
Liver metastasis^a^	Present	0.036	1.655	1.034–2.651
Bone metastasis^a^	Present	0.049	1.503	1.001–2.256
Brain metastasis^a^	Present	0.963	1.020	0.445–2.336
Contralateral renal metastasis^a^	Present	0.349	0.577	0.182–1.826
Local recurrence^a^	Present	0.836	1.085	0.501–2.347
Second‐line therapy	Cabozantinib	0.582	1.126	0.738–1.720
IMDC risk^a^	Favorable	Reference		
	Intermediate	0.299	1.570	0.670–3.679
	Poor	0.002	3.883	1.648–9.151
eGFR slope	≥ 0 mL/min/1.73 m^2^ per year	Reference		
	< 0 and ≥ −9.7 mL/min/1.73 m^2^ per year	0.926	0.976	0.589–1.618
	< −9.7 mL/min/1.73 m^2^ per year	0.128	1.452	0.898–2.349
eGFR^a^	≥ 60 mL/min/1.73 m^2^	Reference		
	< 60 and ≥ 30 mL/min/1.73 m^2^	0.716	1.085	0.699–1.685
	< 30 mL/min/1.73 m^2^	0.005	2.390	1.293–4.419

Abbreviations: CI, confidence interval; eGFR, estimated glomerular filtration rate; IMDC, International Metastatic RCC Database Consortium; IO, immuno‐oncology; TKI, tyrosine kinase inhibitor.

^a^ These variables were evaluated at the time of second‐line therapy initiation.

### 3.4 Associations Between Renal Function and Oncological Outcomes

**FIGURE 3:**
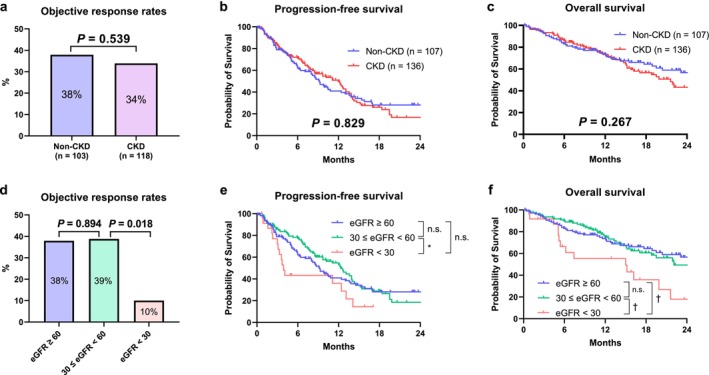
Associations between renal function and oncological outcomes. The objective response rates to second‐line TKI therapy were compared using the Fisher's exact test or chi‐squared test (a and d) between patients with and without chronic kidney disease (CKD) using the chi‐squared test. Progression‐free survival (b and e) and overall survival (c and f) were evaluated using the Kaplan–Meier method and compared using the log‐rank test. These outcomes were calculated from the date of second‐line TKI therapy initiation to the date of the first event or last follow‐up. eGFR, estimated glomerular filtration rate; n.s., not significant; *p < 0.05; ^†^ *p* < 0.01.

Of the 243 patients, the best response with second‐line TKI therapy could be assessed in 221 patients. The objective response rate (ORR) in all patients was 36%. The ORRs were not significantly different between patients with and without CKD (Figure 3a; 34% vs. 38%, respectively, *p* = 0.539). The ORRs were not significantly different between the eGFR ≥ 60 and 30 ≤ eGFR < 60 groups (Figure 3d; 38% vs. 39%, respectively, *p* = 0.894), whereas the ORR of the eGFR < 30 group was significantly lower than that of the 30 ≤ eGFR < 60 group (Figure 3d; 10% vs. 39%, respectively, *p* = 0.018).

PFS and OS were not significantly different between patients with and without CKD (Figure 3b,c; *p* = 0.829 and *p* = 0.267, respectively). PFS was not significantly different between the eGFR ≥ 60 and 30 ≤ eGFR < 60 groups (Figure 3e; *p* = 0.706), whereas the eGFR < 30 group had significantly shorter PFS than the 30 ≤ eGFR < 60 group (Figure 3e; *p* = 0.014). OS was not significantly different between the eGFR ≥ 60 and 30 ≤ eGFR < 60 groups (Figure 3f; *p* = 0.727), whereas the eGFR < 30 group had significantly shorter OS than the eGFR ≥ 60 and 30 ≤ eGFR < 60 groups (Figure 3f; *p* = 0.007 and *p* = 0.007, respectively).

The univariable analyses demonstrated that eGFR < 60 and ≥ 30 mL/min/1.73 m^2^ had no effects on shorter PFS and OS (Tables 2 and 3; *p* = 0.722 and *p* = 0.716, respectively), while eGFR < 30 mL/min/1.73 m^2^ was significantly associated with shorter PFS and OS (Tables 2 and 3; *p* = 0.041 and *p* = 0.005, respectively). The multivariable analyses indicated that eGFR < 30 mL/min/1.73 m^2^ had no effects on shorter PFS (Table 4; *p* = 0.123), whereas eGFR < 30 mL/min/1.73 m^2^ was independently and significantly associated with shorter OS (Table 5; *p* = 0.049).

**TABLE 4:** Multivariable analysis for progression‐free survival.

	Factor	*p*	Hazard ratio	95% CI
Prior nephrectomy	Performed	0.159	0.759	0.517–1.114
Histopathological type	Non‐clear cell	0.006	1.803	1.179–2.756
Sarcomatoid component	Present	0.021	1.903	1.104–3.282
First‐line therapy	IO plus TKI	0.037	1.652	1.032–2.643
Reason for first‐line treatment discontinuation	Progressive disease	0.062	1.522	0.979–2.367
Lactate dehydrogenase^a^	Continuous	0.319	1.000	1.000–1.001
Albumin^a^	Continuous	0.890	0.975	0.682–1.395
C‐reactive protein^a^	Continuous	0.049	1.049	1.000–1.100
Liver metastasis^a^	Present	0.393	1.208	0.783–1.864
IMDC risk^a^	Favorable	Reference		
	Intermediate	0.213	1.575	0.770–3.221
	Poor	0.028	2.606	1.111–6.110
eGFR^a^	≥ 60 mL/min/1.73 m^2^	Reference		
	< 60 and ≥ 30 mL/min/1.73 m^2^	0.668	0.920	0.630–1.345
	< 30 mL/min/1.73 m^2^	0.123	1.601	0.881–2.911

Abbreviations: CI, confidence interval; eGFR, estimated glomerular filtration rate; IMDC, International Metastatic RCC Database Consortium; IO, immuno‐oncology; TKI, tyrosine kinase inhibitor.

^a^ These variables were evaluated at the time of second‐line therapy initiation.

**TABLE 5:** Multivariable analysis for overall survival.

	Factor	*p*	Hazard ratio	95% CI
Prior nephrectomy	Performed	0.121	0.680	0.417–1.108
Histopathological type	Non‐clear cell	0.047	1.658	1.006–2.732
Sarcomatoid component	Present	0.018	2.145	1.142–4.031
First‐line therapy	IO plus TKI	0.029	1.911	1.070–3.411
Reason for first‐line treatment discontinuation	Progressive disease	0.008	2.348	1.245–4.429
Albumin^a^	Continuous	0.006	0.517	0.325–0.825
C‐reactive protein^a^	Continuous	0.026	1.061	1.007–1.118
Liver metastasis^a^	Present	0.429	1.238	0.729–2.105
Bone metastasis^a^	Present	0.116	1.453	0.911–2.317
IMDC risk^a^	Favorable	Reference		
	Intermediate	0.480	1.482	0.498–4.412
	Poor	0.452	1.590	0.475–5.329
eGFR^a^	≥ 60 mL/min/1.73 m^2^	Reference		
	< 60 and ≥ 30 mL/min/1.73 m^2^	0.788	1.068	0.661–1.725
	< 30 mL/min/1.73 m^2^	0.049	1.924	1.004–3.688

Abbreviations: CI, confidence interval; eGFR, estimated glomerular filtration rate; IMDC, International Metastatic RCC Database Consortium; IO, immuno‐oncology; TKI, tyrosine kinase inhibitor.

^a^ These variables were evaluated at the time of second‐line therapy initiation.

## 4 Discussion

To the best of our knowledge, this is the first study to investigate the effects of renal impairment and eGFR slope during first‐line IO combination therapy on oncological outcomes in mRCC patients treated with second‐line TKI therapy. Although severe renal impairment (eGFR < 30 mL/min/1.73 m^2^) did not significantly increase the TKI‐related AE rates or shorten PFS in the present study, it did significantly and negatively impact OS. Moderate renal impairment and rapid renal function decline during first‐line IO combination therapy had no effects on oncological outcomes. These findings suggest that TKIs can be safely used as a second‐line treatment after first‐line IO combination therapy in patients with CKD without sacrificing oncological outcomes, except for patients with severe renal impairment.

Because of the high number of mRCC patients with renal impairment [17], who are typically excluded from clinical trials [18], it is crucial to investigate the efficacy of TKIs in patients with CKD. In the present study, impaired renal function was not associated with shorter PFS in patients treated with second‐line TKIs. These results are consistent with those of previous studies. Macfarlane et al. evaluated the impact of CKD on oncological outcomes in 529 mRCC patients treated with second‐line TKIs, including sunitinib (*n* = 323), sorafenib (*n* = 165), and bevacizumab (*n* = 41). They demonstrated that CKD did not contribute to a lower ORR or poor OS [11]. Similarly, Masini et al. investigated the effect of CKD on oncological outcomes in 229 mRCC patients treated with first‐line pazopanib, showing that impaired renal function did not adversely affect the efficacy of pazopanib, including the response rates, PFS, and OS [12]. Although these data indicate that renal impairment does not affect TKI effectiveness, the results contradict a recent insight into the interaction between cancers and renal function. Because CKD induces immune dysfunction, reduced DNA repair ability, chronic inflammation, and oxidative stress [19], CKD is reportedly related to a 20%–48% increase in cancer‐specific mortality across cancer subtypes [20, 21]. The reasons behind this discrepancy remain unclear because of the lack of RCC‐related data. The starting dose of TKIs in patients with renal impairment was reportedly lower than that in those with normal renal function [22], and both the starting dose and relative dose intensity of TKIs may influence oncological outcomes. Unfortunately, however, our database does not contain this information. Furthermore, although our results showed that severe renal impairment (eGFR < 30 mL/min/1.73 m^2^) was significantly associated with shorter OS, the small number of patients in this CKD stage (*n* = 24, 9.9%) prevents us from making a definitive conclusion. The evidence focusing on patients with severe renal failure is limited. Thus, further clinical and basic research is needed to clarify the association between CKD and oncological outcomes in mRCC.

Data regarding TKI safety in patients with renal impairment are limited, especially for cabozantinib and axitinib, which were mainly used in the present study (cabozantinib, *n* = 118 [49%]; axitinib, *n* = 92 [38%]). Chen et al. reported that the pharmacokinetics and safety of axitinib in patients with renal impairment, including severe renal impairment (creatinine clearance 15–29 mL/min) and end‐stage renal disease (creatinine clearance < 15 mL/min), were similar to those in patients with normal kidney function [23]. However, a Japanese multicenter cohort study that included 477 mRCC patients treated with axitinib showed that the rates of hypertension and renal and urinary disorders of any grade in patients with eGFR < 30 mL/min/1.73 m^2^ were significantly higher than those in the other groups [24]. For cabozantinib, a pharmacological study by Nguyen et al. demonstrated that the geometric least‐squared mean ratios for the area under the curve of plasma cabozantinib from time zero to infinity in patients with mild‐to‐moderate renal impairment were 6%–30% higher than those in patients with normal renal function [25]. These results indicate that dose reduction is necessary in patients with CKD, regardless of axitinib and cabozantinib being primarily metabolized in the liver by cytochrome CYP3A4 and renal excretion being limited. The starting dose of TKIs in patients with renal impairment was reportedly lower than that in those with normal renal function [22], and both the starting dose and relative dose intensity of TKIs may influence the incidence of AEs. Thus, the rates of TKI dose reduction could potentially have contributed to our results. However, unfortunately, the present study did not have this information on second‐line TKI doses. Thus, further studies are warranted to elucidate the relationship between safety and renal impairment.

IO therapies can cause not only acute kidney injury, but also gradual renal function decline over time [14, 26], with a rapid renal function decline being reportedly associated with an increased risk of cancer‐specific mortality in several malignancies [15, 16]. In the present study, although 61 (25%) patients experienced rapid renal function decline (< −9.7 mL/min/1.73 m^2^ per year) during first‐line IO combination therapy, it was not found to be significantly associated with poor oncological outcomes. Kuo et al. conducted a retrospective longitudinal cohort study that included 61 988 older participants, revealing that older individuals with rapid renal function decline had a higher cancer mortality rate across cancer subtypes [16]. Moreover, Gadalean et al. investigated the impact of rapid renal function decline on all‐cause mortality in patients with hepatocellular carcinoma treated with percutaneous injection therapy. They observed that a rapid decline in eGFR significantly contributed to a higher mortality risk, independent of other risk factors [15]. However, the causative mechanisms are not fully understood. Several studies have shown that a rapid renal function decline, even with relatively well‐preserved renal function at baseline, was associated with cardiovascular and all‐cause mortality in the general population and patients with diabetes mellitus and/or hypertension [27, 28, 29]. Thus, non‐cancer‐specific death potentially considerably contributed to the results of these previous studies. Moreover, our study solely focused on patients with metastatic disease, unlike the previous studies. Therefore, the effects of a rapid renal function decline may have been attenuated. A further prospective study is warranted to investigate the association between a rapid renal function decline and oncological outcomes in mRCC patients.

The present study included 53 (22%) patients with non‐clear cell RCC. Although the efficacy of TKIs was demonstrated in phase 3 trials focusing solely on clear cell RCC, the evidence in non‐clear cell RCC remains limited. The Michinoku Japan Urological Cancer Study Group reported that the efficacy of molecular‐targeted agents as first‐line therapy was comparable between patients with metastatic clear cell RCC and those with non‐clear cell RCC [30]. However, in contrast, the present study found that patients with non‐clear cell RCC had significantly shorter PFS and OS compared to those with clear cell RCC (Tables 4 and 5). The reason for this discrepancy remains unclear. Given the small sample sizes in both previous and present studies [30], further research with a sufficient number of patients is warranted.

The present study has several limitations. First, the retrospective study design prevented us from drawing definitive conclusions. We were unable to control for selection bias and other immeasurable confounders. Second, this work included a relatively small number of patients. Third, TKI dose information was not available. The reduced dose could possibly affect the AE rates and oncological outcomes. Fourth, information on comorbidities such as hypertension and diabetes mellitus is lacking. Furthermore, the cause of renal function decline during first‐line IO combination therapy remains unclear. Finally, we measured eGFR at only two time points: the time of first‐line therapy initiation and the time of second‐line therapy initiation. This might have resulted in an under‐ or overestimated eGFR slope during first‐line IO combination therapy.

In conclusion, TKIs can be safely used as a second‐line treatment method after first‐line IO combination therapy in mRCC patients with CKD without sacrificing oncological outcomes, except for in patients with severe renal impairment.

## Author Contributions

**Naoki Fujita:** conceptualization, formal analysis, funding acquisition. **Yuto Matsushita:** conceptualization, data curation, methodology, project administration, writing – original draft, writing – review and editing. **Takahiro Kojima:** conceptualization, data curation, methodology, project administration, writing – review and editing. **Yukari Bando:** data curation, writing – review and editing. **Takahiro Osawa:** data curation, writing – review and editing. **Tomokazu Sazuka:** data curation, writing – review and editing. **Keisuke Goto:** data curation, writing – review and editing. **Kazuyuki Numakura:** data curation, writing – review and editing. **Kazutoshi Yamana:** data curation, writing – review and editing. **Shuya Kandori:** data curation, writing – review and editing. **Yoshihide Kawasaki:** data curation, writing – review and editing. **Takuma Kato:** data curation, writing – review and editing. **Makito Miyake:** data curation, writing – review and editing. **Kazutoshi Fujita:** data curation, writing – review and editing. **Kosuke Ueda:** data curation, writing – review and editing. **Hajime Tanaka:** data curation, writing – review and editing. **Ryotaro Tomida:** data curation, writing – review and editing. **Hiroshi Kitamura:** conceptualization, data curation, methodology, project administration, writing – review and editing. **Hideaki Miyake:** conceptualization, data curation, methodology, project administration, writing – review and editing. **Shingo Hakakeyama:** data curation, writing – review and editing.

## Disclosure

Approval of the Research Protocol by an Institutional Review Board and Registration Number: This study was conducted under the Japanese Urological Oncology Group (JUOG) framework and approved by the ethics review board at Hamamatsu University School of Medicine (Approval No. 22‐008), followed by those of all 34 participating institutions in Japan.

## Consent

The need to obtain informed consent was waived because of the retrospective design; however, the ability to opt out was provided through the websites of all institutions.

## Conflicts of Interest

Takahiro Osawa received honoraria from Takeda and Ono. Tomokazu Sazuka received honoraria from Takeda and BMS. Shingo Hatakeyama received honoraria from MSD. Kazutoshi Fujita received honoraria from Ono, Bristle, MSD, Pfizer, Merck, and Takeda and a grant from Pfizer. Hajime Tanaka received honoraria from MSD. Hiroshi Kitamura received honoraria from Bristle, Merck, MSD, and Takeda. Hideaki Miyake received honoraria from Takeda and MSD. The other authors declare no conflicts of interest. The funders had no role in the study design, data collection, analysis, interpretation, manuscript writing, or decision to publish the results. Kazutoshi Fujita, Hajime Tanaka, Hideaki Miyake, and Shingo Hatakeyama are the Editorial Board members of International Journal of Urology and the co‐authors of this article. To minimize bias, they were excluded from all editorial decision‐making related to the acceptance of this article for publication.

## Supporting information


**Figure S1.** Associations between renal function decline during first‐line therapy and oncological outcomes. The estimated glomerular filtration rate (eGFR) was compared at the time of first‐line therapy initiation and second‐line therapy initiation using the Mann–Whitney *U* test (a–d). Progression‐free survival (PFS) was evaluated using the Kaplan–Meier method and compared using the log‐rank test (e, f). PFS was calculated from the date of second‐line tyrosine kinase inhibitor (TKI) therapy initiation to the date of the first event or last follow‐up. I‐O, immuno‐oncology; n.s., not significant.
