# Supplementary figures and images for: Impact of Impaired Renal Function on the Efficacy and Safety of Second‐Line Tyrosine Kinase Inhibitor Therapy After First‐Line Immuno‐Oncology Combination Therapy in Metastatic Renal Cell Carcinoma: A Japanese Multicenter Retrospective Study
Source: Int J Urol. 2025 Jul 4;32(10):1506–17. doi: 10.1111/iju.70172 (PMC12503203; doi:10.1111/iju.70172)

**Fig. S1**

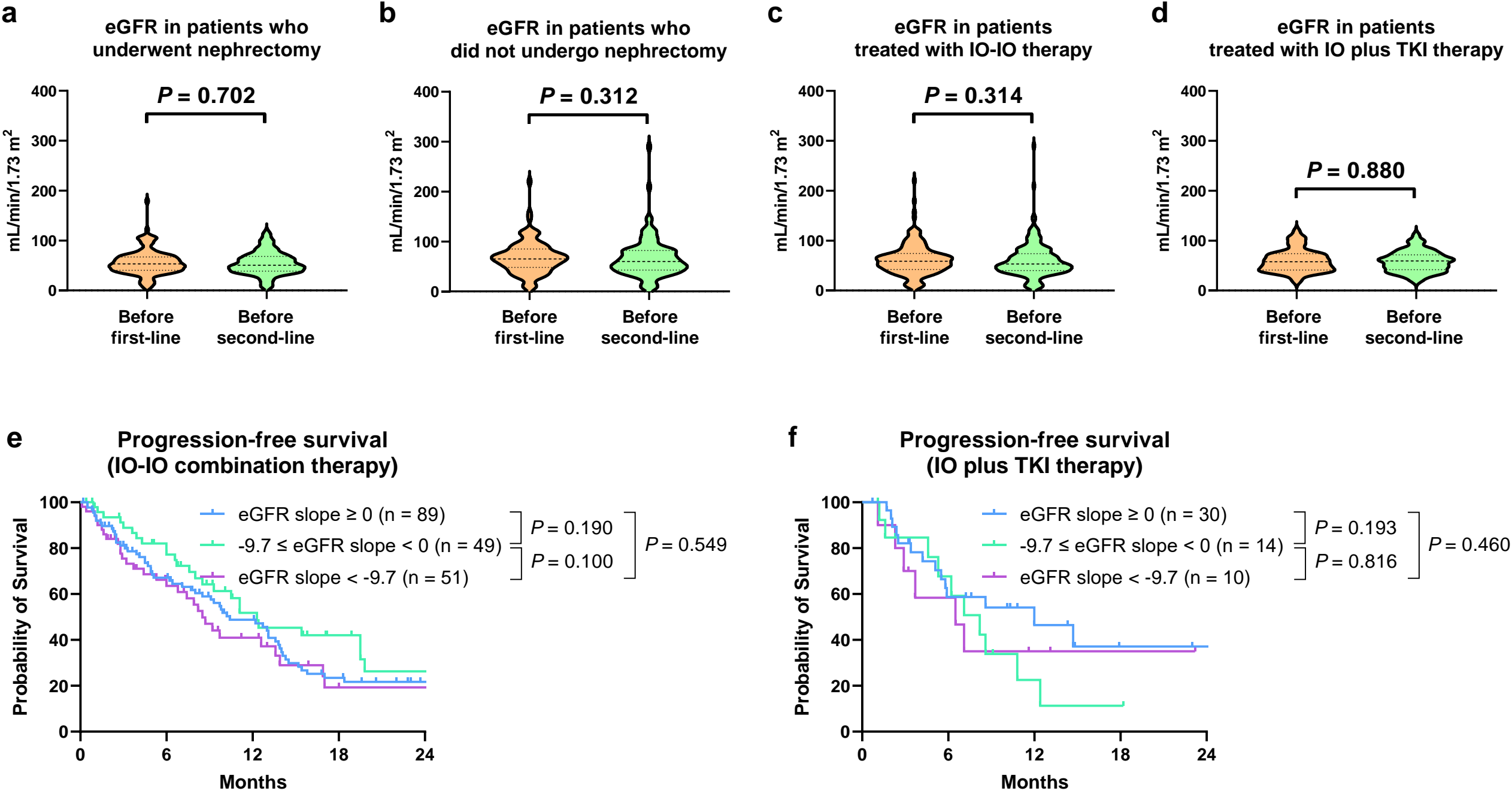

Supplement: Supplementary file 1 — Figure S1. Associations between renal function decline during first‐line therapy and oncological outcomes. The estimated glomerular filtration rate (eGFR) was compared at the time of first‐line therapy initiation and second‐line therapy initiation using the Mann–Whitney U test (a–d). Progression‐free survival (PFS) was evaluated using the Kaplan–Meier method and compared using the log‐rank test (e, f). PFS was calculated from the date of second‐line tyrosine kinase inhibitor (TKI) therapy initiation to the date of the first event or last follow‐up. I‐O, immuno‐oncology; n.s., not significant. [file IJU-32-1506-s001.pdf]
